# The Super-Donor Phenomenon in Fecal Microbiota Transplantation

**DOI:** 10.3389/fcimb.2019.00002

**Published:** 2019-01-21

**Authors:** Brooke C. Wilson, Tommi Vatanen, Wayne S. Cutfield, Justin M. O'Sullivan

**Affiliations:** ^1^The Liggins Institute, University of Auckland, Auckland, New Zealand; ^2^The Broad Institute of MIT and Harvard, Cambridge, MA, United States

**Keywords:** fecal microbiota transplantation (FMT), super-donor, microbial dysbiosis, *clostridium difficile* infection (CDI), inflammatory bowel disease (IBD)

## Abstract

Fecal microbiota transplantation (FMT) has become a highly effective bacteriotherapy for recurrent *Clostridium difficile* infection. Meanwhile the efficacy of FMT for treating chronic diseases associated with microbial dysbiosis has so far been modest with a much higher variability in patient response. Notably, a number of studies suggest that FMT success is dependent on the microbial diversity and composition of the stool donor, leading to the proposition of the existence of FMT super-donors. The identification and subsequent characterization of super-donor gut microbiomes will inevitably advance our understanding of the microbial component of chronic diseases and allow for more targeted bacteriotherapy approaches in the future. Here, we review the evidence for super-donors in FMT and explore the concept of keystone species as predictors of FMT success. Possible effects of host-genetics and diet on FMT engraftment and maintenance are also considered. Finally, we discuss the potential long-term applicability of FMT for chronic disease and highlight how super-donors could provide the basis for dysbiosis-matched FMTs.

## Introduction

The human gut harbors an abundant and diverse microbial community that is as unique to an individual as a fingerprint (Human Microbiome Project Consortium, 2012). Despite the variability between individuals, it is clear that the composition and functionality of the gut microbiota associates with the health of the host, having specialized functions in nutrition, energy metabolism, immune development, and host defense (Thursby and Juge, 2017). The composition of the gut microbiota is shaped by both genetic and environmental influences, through a continual process that may begin *in utero* (Perez-Muñoz et al., 2017) and fluctuates throughout an individual's lifetime (Odamaki et al., 2016). In a healthy adult, the bacterial population within the gut predominantly consists of members from the strictly anaerobic Firmicutes and Bacteroidetes phyla, with minor representations from members of the Proteobacteria and Actinobacteria phyla (Eckburg et al., 2005; Ley et al., 2008).

Because no two gut microbiomes are identical, the definition of what comprises a healthy gut microbiome from an inventory standpoint remains unclear (Human Microbiome Project Consortium, 2012). Despite this, it is generally accepted that having a stable and diverse gut community correlates with a healthy intestinal state (Lloyd-Price et al., 2016). An alteration to the microbiota that is associated with negative functional outcomes on gut physiology, such as localized inflammation or disturbed metabolic processing, is known as gut dysbiosis (Petersen and Round, 2014). Typically, gut dysbiosis is characterized by a low microbial diversity (Kriss et al., 2018).

Observations of microbial dysbioses are increasingly being associated with a broad range of human diseases, including allergies (Penders et al., 2007; Bunyavanich et al., 2016), asthma (Arrieta et al., 2015), inflammatory bowel disease (IBD) (Fujimoto et al., 2013; Gevers et al., 2014; Takahashi et al., 2016; Nishino et al., 2018), irritable bowel syndrome (IBS) (Liu et al., 2017), obesity (Schwiertz et al., 2010), and cardiovascular disease (Cui et al., 2017; Jie et al., 2017). However, evidence that the dysbiosis is causal in the development of these conditions remains difficult to establish, in all but a few cases. The use of germ-free mice, which are born and raised in a sterile environment, play a pivotal role in demonstrating causative associations between the gut microbiota and disease (Balish and Warner, 2002; Bäckhed et al., 2004; Berer et al., 2011). In the case of obesity, the metabolic phenotype of the donor, be it lean or obese, can be recapitulated by a fecal microbiota transfer into germ-free mice (Ridaura et al., 2013). Germ-free conditions have also been found to be protective against the development of colitis and ileitis in IBD-like mouse models with disease transmission only occurring after transfer of a dysbiotic gut microbiota (Sellon et al., 1998; Schaubeck et al., 2016).

The mounting evidence of a causal role of the gut microbiota in multiple disease conditions has led to the development of targeted therapeutic approaches designed to alter the microbial composition. Among these, fecal microbiota transplantation (FMT) has consistently demonstrated a capability to overcome dysbiosis associated with a number of conditions through a profound sustained effect on the gut microbiome (e.g., Weingarden et al., 2015; Broecker et al., 2016; Kumar et al., 2017; Moss et al., 2017). FMT is considered an unrefined form of bacteriotherapy that utilizes the diverse microbial gut community of a healthy donor. Typical routes of administration to FMT recipients include endoscopic delivery (Mattila et al., 2012), naso-intestinal tube delivery (Tian et al., 2017), retention enemas (Lee et al., 2014), or capsule ingestion (Youngster et al., 2014).

The definition of FMT success is primarily based on a positive clinical response in the recipient. However, from a microbiological perspective, FMT success can also be defined by a shift in the gut microbiome profile of an individual toward that of the donor. We argue that FMT success can be considered to be a two-step process; first requiring the transplanted microbiome to engraft within the new host and augment the local commensal community, after which clinical improvement may be observed.

**Figure 1:**
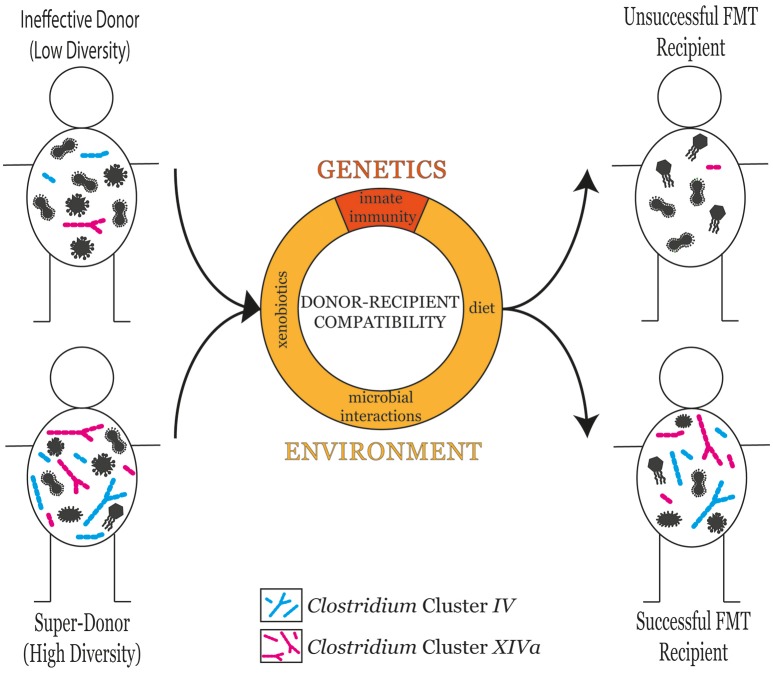
The microbial diversity of the donor is a good predictor of FMT success in the recipient. However, donor-recipient compatibility also plays an influential role in determining FMT success. Donor-recipient compatibility can stem from genetic factors such as differences in innate immune responses, or environmental factors including diet, xenobiotic exposure, and microbial interactions.

The selection of an appropriate stool donor is a key component in FMT success (Vermeire et al., 2016). Donors are clinically screened to ensure they do not harbor any transmissible pathogens or disease (Kelly et al., 2015). A detailed list of donor selection guidelines can be found in the recently published evidence-based report on FMT in clinical practice (Cammarota et al., 2017). Donors are typically described as being either effective or ineffective with regards to their ability to contribute to FMT success. Comparing the gut microbiota profiles of different donors has revealed that microbial diversity is a reliable predictor for FMT success (Kump et al., 2018). However, a variety of additional factors, both genetic and environmental, are also known to influence FMT success (Figure 1).

Recently, the term “super-donor” has been proposed to describe donors whose stool results in significantly more successful FMT outcomes than the stool of other donors. The purpose of this review is to explore evidence for the phenomenon of FMT super-donors and other factors that can contribute to treatment success, particularly focusing on the gut microbiota characterization that has been performed on donors. We discuss the concept of keystone species as predictors of FMT success and consider the possible influence of host-genetics and diet on FMT engraftment and maintenance. Finally, we will suggest a rationale for abandoning the “one stool fits all” approach.

## FMT for Recurrent *Clostridium difficile* Infection

The ingestion of human stool for health-related purposes was first documented by Chinese herbal doctors in the fourth century (de Groot et al., 2017). In recent years, FMT has been widely and effectively used for the treatment of recurrent *Clostridium difficile* infections (CDI) in patients that are non-responsive to antibiotic therapy (van Nood et al., 2013; Cammarota et al., 2015; Lee et al., 2016; Kao et al., 2017). *C. difficile* is an opportunistic gut pathogen that is suppressed in healthy individuals by the commensal gut microbiota (Borriello, 1990). However, when the diversity of the gut microbiota is reduced, e.g., after a course of antibiotics, colonization resistance of the commensal microbiota is disturbed (Leffler and Lamont, 2015). *C. difficile* then has the potential to proliferate undeterred, producing enterotoxins leading to intestinal inflammation and diarrhea (Warny et al., 2005). Perhaps rather counterintuitively, CDI is treated in the first instance with antibiotics which cure around 80% of cases (Fekety et al., 1997). However, 20% of individuals will experience recurrent CDI after antibiotic therapy (Leffler and Lamont, 2015). FMT is a novel treatment approach for recurrent CDI that acts to restore the commensal gut microbiota and in turn re-establish colonization resistance to inhibit the growth of *C. difficile* (Eiseman et al., 1958).

A recent systematic review and meta-analysis of FMT for the treatment of CDI reported a primary cure rate of 92% across 30 case series and seven randomized control trials (Quraishi et al., 2017). Microbial analyses carried out on CDI patients before and after FMT have confirmed FMT is rapidly capable of restoring microbial diversity in patients to donor-like proportions (Song et al., 2013; Shankar et al., 2014; Kelly et al., 2016; Staley et al., 2016; Khanna et al., 2017; Kellingray et al., 2018). The choice of donor, be it a relative, spouse, or anonymous volunteer, does not appear to influence the clinical efficacy of FMT (Kassam et al., 2013). Similarly, no donor-specific effects were found in a large cohort study comprising 1999 CDI patients and 28 FMT donors (Osman et al., 2016). Overall, FMT appears to be a safe and effective treatment for microbial restoration in situations where the overgrowth of a particular pathogen has led to a reduction in the diversity and abundance of the commensal organism population (i.e., severe dysbiosis).

## FMT for Chronic Diseases Associated With Intestinal Dysbiosis

Encouraged by the overwhelming success of FMT in the resolution of recurrent CDI, researchers have begun to investigate the therapeutic potential of FMT for a broad range of other diseases associated with less severe forms of intestinal dysbiosis. Among these exploratory studies, FMT for the treatment of IBD has featured heavily (systematically reviewed Paramsothy et al., 2017b). However, FMT has also been trialed in several other gastrointestinal disorders [IBS (Pinn et al., 2014; Holvoet et al., 2017, 2018; Mizuno et al., 2017; Aroniadis et al., 2018; Halkjær et al., 2018; Johnsen et al., 2018), constipation (Tian et al., 2017; Ding et al., 2018), allergic colitis (Liu et al., 2017)] and for various liver (Kao et al., 2016; Bajaj et al., 2017; Philips et al., 2017; Ren et al., 2017), blood (Kakihana et al., 2016; Spindelboeck et al., 2017; DeFilipp et al., 2018), metabolic (Vrieze et al., 2012; Kootte et al., 2017), and neurological conditions (He et al., 2017a; Kang et al., 2017; Makkawi et al., 2018). Compared with CDI, the clinical efficacy of FMT for these more chronic diseases has so far been modest with a much higher variability in patient response which likely reflects the multi-faceted etiology of these disorders.

### FMT for Inflammatory Bowel Disease (IBD): The Emergence of the FMT Super-Donor

IBD encompasses both Crohn's disease and ulcerative colitis; two debilitating disorders characterized by chronic relapsing inflammation of the intestinal mucosa (Gajendran et al., 2018). In contrast to CDI, there is no evidence that IBD results from an overgrowth of one specific pathogen. Rather, the disease is likely brought on by complex interactions involving the host's genetics, immune system, and gut microbiota (Ni et al., 2017). Both Crohn's disease and ulcerative colitis are broadly characterized by a reduced diversity of the gut microbiota with lower relative abundances of the Bacteroidetes and Firmicutes phyla and higher proportions of Proteobacteria (Manichanh et al., 2006; Frank et al., 2007; Sokol et al., 2008; Walker et al., 2011; Gevers et al., 2014; Machiels et al., 2014; Nishino et al., 2018). A specific reduction in the abundance of butyrate-producing bacterial species, particularly *Faecalibacterium prausnitzii*, has been observed for both Crohn's disease and ulcerative colitis (Fujimoto et al., 2013; Lopez-Siles et al., 2015; Takahashi et al., 2016). Meanwhile, for Crohn's disease, an increase in a pro-inflammatory form of *Escherichia coli* has also been reported (Darfeuille-Michaud et al., 2004; Martin et al., 2004; Baumgart et al., 2007).

The first successful case report of an FMT for the treatment of IBD was published in 1989 when a male with refractory ulcerative colitis achieved clinical remission for 6 months following a retention enema with healthy donor stool (Bennet and Brinkman, 1989). Subsequently, a large number of FMT studies have been conducted on IBD patients with variable clinical outcomes, remission rates, and longevity of effect (Zhang et al., 2013; Cui et al., 2015; Moayyedi et al., 2015; Rossen et al., 2015; Suskind et al., 2015; Vaughn et al., 2016; Vermeire et al., 2016; Costello et al., 2017; He et al., 2017b; Nishida et al., 2017; Paramsothy et al., 2017a; Goyal et al., 2018; Kump et al., 2018). Recently, Paramsothy et al. performed a systematic review and meta-analysis of 53 studies (four RCT, 30 cohort, 19 case studies) of FMT in IBD patients (Paramsothy et al., 2017b). Avoiding publication bias, their analysis of cohort studies revealed FMT was more effective at inducing remission in Crohn's disease patients when compared to patients with ulcerative colitis (52 vs. 33%, respectively). With regard to ulcerative colitis, a larger number of FMT infusions and a lower gastrointestinal tract administration were associated with improved rates of remission.

In contrast to studies of CDI, FMT studies conducted on IBD patients have frequently identified differential recipient responses that have been associated with variability in the donor stool (Khanna, 2018). Currently, the stool used for FMT is not standardized in terms of donor selection (related vs. unrelated), preparation (fresh vs. frozen, aerobic vs. anaerobic), or the dose that is administered (single vs. multiple doses) (Kelly et al., 2015). While inconsistencies in FMT protocols make it difficult to compare different studies, there is a large degree of variability in clinical responses to FMT between recipients who have been subjected to the same study design. It is unfortunate that information on a recipient's genetic background or dietary intake is not yet routinely assessed, particularly given that some instances of IBD have an underlying genetic component (de Lange et al., 2017). Due to the lack of genetic information, investigators have instead focused on the donor-dependent effect and proposed the existence of so called super-donors to explain the variation in recipient responses.

The first study to record the super-donor effect was a randomized control trial that was investigating the efficacy of FMT for inducing clinical remission in patients with ulcerative colitis (Moayyedi et al., 2015). Moayyedi et al. assigned 75 patients with active disease to weekly enemas containing either fecal material or water (placebo) for a period of 6 weeks. FMT was shown to be superior to the placebo, resulting in significantly higher rates of endoscopic and clinical remission, albeit of modest effect (24 vs. 5%, respectively), after 7 weeks. Of the nine patients who entered remission, seven had received FMT from the same donor. Thus, it was argued that FMT success was donor-dependent.

Currently, it is not possible to predict the clinical efficacy of a donor before FMT in IBD patients. It has been suggested that remission rates could be improved by pooling donor's stool together, limiting the chances a patient will receive only ineffective stool (Kazerouni and Wein, 2017). This stool pooling approach was recently investigated on an Australian cohort of 85 mild to moderate ulcerative colitis patients, in the largest randomized control trial of FMT for IBD to date (Paramsothy et al., 2017a). Rather than receiving FMT from just one donor, patients in the treatment arm were administered a stool mixture that contained contributions from up to seven different donors with the hope that donor-dependent effects could be homogenized. In addition to this, a far more intensive dosing program was adopted with an initial FMT delivered by colonoscopy that was followed by fecal enemas, five times a week for 8 weeks. Despite the multi-donor and intensive dosing approach, Paramsothy et al. achieved post-FMT remission rates (FMT, 27% vs. placebo, 8%, *p* = 0.02) that were similar to those reported previously (Moayyedi et al., 2015; Rossen et al., 2015). Notably, however, both clinical and endoscopic remission were required for primary outcome achievement in this study (Paramsothy et al., 2017a), whereas previous studies have mostly focused on either endoscopic or clinical remission rates alone (Ishikawa et al., 2017; Nishida et al., 2017). The pooled stool mixture was demonstrated to have higher microbial diversity than individual stool alone based on OTU count and phylogenetic diversity measures. Subsequent analysis of the different stool batches discovered that one donor appeared to exhibit a super-donor effect. Specifically, patients that received FMT batches that contained stool from this one donor exhibited a higher remission rate than those whose FMT batches did not include the super-donor (37 vs. 18%, respectively) (Paramsothy et al., 2017a).

### FMT for Other Disorders: Is There Also a Super-Donor Effect?

Evidence of FMT super-donors in other disorders outside of IBD is currently lacking. Case series and reports limit the capacity to identify super-donor effects because of limited sample sizes. However, despite the lack of large cohort studies, several studies have hinted at the possibility of a donor-dependent effect on FMT outcome (Vrieze et al., 2012; Kootte et al., 2017; Mizuno et al., 2017). For example, in a short-term FMT pilot trial on 18 middle-aged men with metabolic syndrome, FMTs from lean donors (allogenic FMT) were found to correspond with a 75% increase in insulin sensitivity and a greater diversity of intestinal bacteria in the recipient compared to autologous FMTs (recipient-derived) (Vrieze et al., 2012). It was later noted that the patients who experienced a more robust improvement of insulin sensitivity post-FMT had all been in receipt of the same donor. In a subsequent study on 38 Caucasian men with metabolic syndrome, lean donor FMT also resulted in a significant improvement in peripheral insulin sensitivity at 6 weeks. However, this effect was lost by the 18 week follow up (Kootte et al., 2017). For the allogenic FMT, 11 lean donors were used, seven of which were used for more than one recipient. Whilst donor-dependent effects were not reported, the authors noted that the “multiple fecal donors might explain the transient and variable effects seen in the allogenic group.” As FMT research in this field progresses from small-scale case series to larger-scale randomized placebo controlled clinical trials, it remains to be seen whether the super-donor phenomenon generalizes to other conditions outside of IBD.

## Microbial and Metabolic Profiling: Can We Characterize a Super-donor?

To shed light on the varying patient responses to FMT and uncover any donor-dependent effects, a number of studies have carried out microbial profiling on donors and recipients before and after FMT (Vrieze et al., 2012; Moayyedi et al., 2015; Rossen et al., 2015; Vaughn et al., 2016; Vermeire et al., 2016; Bajaj et al., 2017; Fuentes et al., 2017; Mizuno et al., 2017; Paramsothy et al., 2017a; Kump et al., 2018). Despite a lack of large-cohort based studies, one key theme has begun to emerge: the donor's microbial diversity has an influential role in the therapeutic success of FMT (Vermeire et al., 2016; Kump et al., 2018).

It has been consistently shown that FMT recipients experience a significant increase in gut microbiota diversity, typically shifting in composition toward the profile of their respective stool donor (Vaughn et al., 2016; Paramsothy et al., 2017b). Those who achieve a clinical response to FMT (responders) typically exhibit a higher microbial diversity than those who do not (non-responders) (Vaughn et al., 2016; Vermeire et al., 2016) (Figure 1). In line with these observations, the microbial diversity of the stool donor has been shown to be one of the most significant factors influencing FMT outcome (Kump et al., 2018). In a Belgian IBD cohort, Vermeire et al. observed significantly higher bacterial richness in donors that produced a clinical response to FMT than those who did not (Vermeire et al., 2016).

A specific microbial signature that correlate with the clinical efficacy of FMT for IBD has also been explored (Moayyedi et al., 2015; Rossen et al., 2015; Vermeire et al., 2016; Fuentes et al., 2017; Nishida et al., 2017; Paramsothy et al., 2017a; Kump et al., 2018). Among the various taxa that have been reported, *Clostridium* clusters *IV* and *XIVa* have consistently been shown to be indicative of a positive patient response to FMT (Rossen et al., 2015; Fuentes et al., 2017; Paramsothy et al., 2017a). *Clostridium* clusters *IV* and *XIVa* are informal groups of bacteria that mostly include genera from the Ruminococcaceae and Lachnospiraceae family, respectively. Specific genera within these *Clostridium* clusters (e.g., *Roseburia, Oscillibacter, Blautia, Dorea*) have been shown to increase in relative abundance in responders following FMT (Moayyedi et al., 2015; Rossen et al., 2015; Vermeire et al., 2016; Paramsothy et al., 2017a). Likewise, stool donors that are rich in specific members of *Clostridium* clusters *IV* and *XIVa* have been found to be predictive of sustained FMT response in IBD patients (Rossen et al., 2015; Fuentes et al., 2017). Notably, the gut microbiome from the super-donor identified by Moayyedi et al. was enriched with Ruminococcaceae and Lachnospiraceae families (Moayyedi et al., 2015).

To further characterize FMT super-donors, metabolic differences between responders and non-responders have been investigated. In particular, an increased production of butyrate by key members within *Clostridium* clusters *IV* and *XIVa* has been associated with prolonged clinical remission in IBD in response to FMT therapy (Fuentes et al., 2017). Butyrate is an important short chain fatty acid (SCFA), produced by bacteria in the gut, with specialized functions in immune modulation and energy provision (Tan et al., 2014). An increased production of butyrate has also been associated with CDI resolution following FMT (Kellingray et al., 2018). Similarly, the butyrate-producing species *Roseburia intestinalis* was found to increase two and a half fold in obese participants given FMT from lean donors (Vrieze et al., 2012).

Collectively, published observations suggest that microbial restoration can lead to alterations in metabolic outputs, which may be responsible for resetting the gut homeostasis in dysbiotic individuals. This is consistent with the idea that the key to FMT success lies in the ability of the donor to transfer high levels of particular keystone species to recipients. For inflammatory conditions, such as IBD and metabolic syndrome, transfer of butyrate-producing taxa may be important for therapeutic restoration. By contrast, donors with high abundances of *Bifidobacterium* may be more effective at treating patients with IBS (Mizuno et al., 2017).

The concept of keystone species was recently employed in a FMT study for recurrent hepatic encephalopathy (rHE) (Bajaj et al., 2017). Frequent exposure to antibiotics causes dysbiosis and decreased relative abundances of SCFA-producing families in rHE patients (Chen et al., 2011). Therefore, a rationalized donor selection approach was adopted in which microbiome data was used to select a donor with the highest relative abundance of families Lachnospiraceae and Ruminococcaceae from the universal stool donor bank, OpenBiome (Bajaj et al., 2017). In total, 10 patients received a 5-day course of broad-spectrum antibiotics followed by a single FMT enema from the selected donor. At 5 months post-FMT, none of the 10 FMT patients had experienced a recurrence of HE, compared to half (5/10) of the control patients who received the current standard of care (lactulose and rifaximin). Gut microbiome profiling revealed that the FMT patients had an enrichment for Ruminococcaceae but not Lachnospiraceae at 20 days post-FMT. Whilst rationalized donor selection is a step in the right direction, these results suggest that microbial enrichment in the donor does not completely guarantee enrichment in the FMT recipient. The forces governing FMT engraftment must therefore not solely be based on donor input.

## Microbial Interactions Influencing FMT Engraftment

FMT engraftment involves the integration or establishment of donor-derived microbial strains into the recipient's gut microbial community. Currently, it appears that the most important factors predicting strain engraftment in FMT are taxonomic identity and strain abundance in both the donor and the recipient prior to FMT (Smillie et al., 2018). Deep metagenomic sequencing enables strain level analysis for tracking microbial alterations and engraftment in the post-FMT gut microbiome. For example, Li et al. demonstrated that new microbial strains from the donor had a higher likelihood of engrafting if the recipient already possessed that species (Li et al., 2016). This led them to suggest that differences in microbiome engraftment between individuals of the same donor may stem from strain incompatibilities between the donor and the FMT recipient. Smillie et al. reported that strains of any given species engrafted in an “all or nothing” manner such that strains were either completely retained or completely replaced by donor strains in the recipient's post-FMT gut microbiome (Smillie et al., 2018). Meanwhile on a community level, FMT recipients harbored a complex mixture of both recipient-derived, donor-derived, and newly-acquired species post FMT (Smillie et al., 2018). Overall, it appears that microbial interactions have a significant part to play in FMT engraftment which helps to explain why dual recipients of a donor FMT do not exhibit identical gut microbiota profiles.

## The Influence of Host Genetics: Immune Response to FMT

Genetics has been estimated to explain 5–10% of the variability in bacterial taxa observed between individuals(Willing et al., 2010; Goodrich et al., 2014, 2016; Wang et al., 2016; Xie et al., 2016; Hall et al., 2017). Among the taxa that have been found to be heritable, the majority have been linked to genes that are involved in innate immunity (Hall et al., 2017). The gut microbiota is known to be intricately connected to the host's immune system through a reciprocal developmental relationship. Specifically, the microbiome is critical for the appropriate development of the immune system, and in turn, the immune system helps modulate the microbiome community through a balance of pro- and anti-inflammatory pathways (Belkaid and Hand, 2014; Vatanen et al., 2016). Therefore, it remains possible that incompatibilities that arise from FMT may be attributable to a heightened immune response to the transplanted microbiota, possibly stemming from an underlying genetic difference between the donor and the recipient.

Taking this into consideration, an immune screening approach was recently investigated in a FMT case study for ulcerative colitis (Ponce-Alonso et al., 2017). To avoid FMT rejection by the immune system, a rectal biopsy was obtained from an ulcerative colitis patient in order to isolate a population of lymphoid cells. These patient-derived lymphoid cells were incubated with different gut microbiota samples isolated from three healthy stool donors. The donor microbiota that resulted in the lowest induction of pro-inflammatory interleukins was subsequently selected for FMT. The FMT proved to be a clinical success for the ulcerative colitis patient. Gut microbiome profiling revealed the ulcerative colitis patient's gut microbiome had become indistinguishable from that of the donor's, indicating highly successful FMT engraftment. Whilst immune screening in this instance led to a successful outcome for the FMT patient, the associated time and costs of running such a screen limit scalability to larger patient populations. Ideally, the development of a quick and simple rectal swab assay to assess immune response would be a much more feasible screening approach moving forward. In any case, the limited literature in this area must be supplemented by larger-scale studies to confirm the importance of immune screening prior to FMT.

## Factors Affecting the Long-Term Effects of FMT

It could be argued that a successful FMT not only requires the transplanted microbiota to engraft within the recipient's gut, but the newly acquired organisms must also be supported for therapeutic benefit to be maintained. Based on longitudinal analyses in patients who have received FMT for recurrent CDI, it is known that FMT-induced microbiota alterations can last anywhere from a few days to a few years after transfer (Weingarden et al., 2015; Broecker et al., 2016; Kumar et al., 2017; Moss et al., 2017). A recent FMT/CDI study by Moss et al. discovered that despite short-term similarity between donor and recipient gut microbiota profiles, concordance was significantly reduced after a year (Moss et al., 2017). In the FMT study by Moayeddi et al. eight of the nine ulcerative colitis patients who were in remission at week seven post-FMT were still in remission a year later with no instances of relapse (Moayyedi et al., 2015). Unfortunately, microbiome analyses were not carried out on these patients during follow-up so it can only be presumed that their transplanted microbiota remained stable.

In addition to underlying genetic differences between donor and the recipient, dietary selection pressures and subsequent antibiotic exposures are also likely to influence the long-term efficacy of FMT therapy. For recurrent CDI, the long-term stability of the FMT is less relevant because clearance of the pathogen and restoration of the commensal population is achieved rapidly. Thus, a gradual drift away from the donor's gut profile is unlikely to result in disease reoccurrence assuming there are no further insults to the commensal gut population. By contrast, the sustainability of the post-FMT microbiota in patients with chronic disease, such as IBD or obesity, may be much more pertinent. This is because the microbial dysbiosis associated with these conditions has not yet been proven in humans to be causal or consequential to disease (Ni et al., 2017). It is more likely that microbial dysbiosis is just one of several factors contributing toward disease progression in these individuals. If so, it may be that FMT provides only temporary relief to patient's symptoms and additional, “top-up FMTs” are required for continual disease management. The optimization of non-invasive FMT delivery approaches, such as capsule-based delivery, will thus be important moving forward.

Supporting the transplanted microbiome through diet could be a beneficial addition to FMT protocols (Thompson et al., 2016). Diet is known to play a significant role in shaping the developing gut microbiome in infancy as well as throughout adulthood (Singh et al., 2017). The diet provides the commensal microbes with substrates required for their proliferation and survival (Koh et al., 2016). It has been shown that a rapid change in diet, such as a switch from an animal-based to an exclusively plant-based diet, can alter the composition of the gut microbiota within 24 h (David et al., 2014). In inflammatory conditions, such as IBD and metabolic syndrome, FMT may act to overcome the initial hurdle in providing patients with therapeutic levels of anti-inflammatory bacteria. Subsequently, diet may be crucial in providing the necessary fiber required to support the growth of SCFA producing bacteria.

## Abandoning the “One Stool Fits All” Approach

Microbial dysbiosis is a blanket term for an unhealthy or imbalanced gut community. As such, the population structure that is considered to represent microbial dysbiosis is variable between different disorders (Duvallet et al., 2017). Moreover, the microbiome deficit of one individual may not necessarily mirror that of another individual and therefore it is not surprising that patients respond differently to FMT. As more FMT-related clinical and microbial data are generated, it is becoming clear that “one stool does not fit all” in the context of treating chronic diseases with microbial dysbiosis. Equally so, the selection of donors based solely on clinical screening guidelines provides no guarantee of FMT success. It appears a patient's response to FMT predominantly depends on the capability of the donor's microbiota to restore the specific metabolic disturbances associated with their particular disease phenotype. If this is true, a donor-recipient matching approach, where a patient is screened to identify the functional perturbations specific to their microbiome, may be the best way forward. The patient could then be matched to a specific FMT donor known to be enriched in taxa associated with the metabolic pathway that needs to be restored. Immune tolerance screening would also be beneficial for reducing the impact of donor-recipient incompatibilities stemming from underlying differences in innate immune responses.

An alternative approach to donor-recipient matching is to administer precision FMTs, which are more akin to a probiotic as opposed to a whole fecal transplant. In addition to the obvious regulatory and consumer advantages inherent in this approach, a precision FMT removes the donor-dependent effects by providing patients with a defined mixture of bacteria that have previously been shown to be beneficial for disease resolution (e.g., enhancing butyrate-production in inflammatory conditions). For example, providing IBD patients with a targeted microbiota-based formulation containing only butyrate-producers would be a logical, safer, and potentially patient preferable alternative to whole fecal transplantation. Precision approaches have so far been trialed in CDI treatment but with mixed results (Petrof et al., 2013; Emanuelsson et al., 2014). It may be that the microbial community structure as a whole plays a more influential role in FMT success than the isolation of critical species alone. Regardless, targeted bacteriotherapy approaches should be investigated for chronic diseases as a way of circumventing the risks associated with administering fecal material.

Whilst much of the FMT literature has focused on bacteria being the therapeutically active agent, the successful resolution of CDI using sterile fecal filtrates has suggested non-bacterial elements might play a more significant role than previously appreciated (Ott et al., 2017). In a preliminary case series, five patients with recurrent CDI were administered a stool solution that had been filtered to remove small particles and bacteria. The fecal filtrate was found to contain bacterial debris, proteins, DNA, antimicrobial compounds, metabolites, and viruses. Notably, a few days post-transfer, all five patients had achieved CDI resolution and remained symptom free for the duration of the study (up to 6 months). Although limited by the number of patients who were treated, this preliminary study demonstrates that resolution of CDI can be achieved by elements other than the live bacterial component of stool. Consistent with this, Zuo et al. recently reported that bacteriophage transfer during FMT was associated with CDI resolution outcomes (Zuo et al., 2018). Similarly, Conceição-Neto et al. suggested the eukaryotic virome was associated with the successful treatment of ulcerative colitis by FMT (Conceição-Neto et al., 2018).

## Conclusion

Despite being reported in the literature as far back as the fourth century, FMT research is still in its infancy, particularly with regards to its mechanism of effect. The lack of large randomized controlled clinical trials of FMT for the treatment of chronic diseases has meant that many observations, such as the existence of FMT super-donors, are not yet robustly supported by empirical evidence. The growing number of small-scale studies, whilst difficult to compare with each other, do however suggest the donor plays an influential role in FMT outcomes for indications outside of CDI. Considerable effort has since been spent in identifying the various factors which contribute to FMT success. In a broad sense, high diversity of the gut microbiota, particularly in the donor, appears to best predict a patient's response to FMT. More specifically, the efficacy of FMT likely depends on the ability of the donor to provide the necessary taxa capable of restoring metabolic deficits in recipients that are contributing toward disease. Further characterization of super-donors will likely result in the development of more refined FMT formulations to help standardize therapy and reduce variability in patient response. In parallel, continued optimization of FMT protocols, including a shift toward capsule-based approaches, will help combat the longevity issues associated with FMT and create a more patient-friendly alternative to current disease management schemes.

## Author Contributions

BW wrote the manuscript. TV and WC commented on the manuscript. JOS directed and contributed to the writing of the manuscript.

### Conflict of Interest Statement

The authors declare that the research was conducted in the absence of any commercial or financial relationships that could be construed as a potential conflict of interest.
